# HIV-1 Tat Interacts with a Kaposi’s Sarcoma-Associated Herpesvirus Reactivation-Upregulated Antiangiogenic Long Noncoding RNA, LINC00313, and Antagonizes Its Function

**DOI:** 10.1128/JVI.01280-19

**Published:** 2020-01-17

**Authors:** Wan-Shan Yang, Ting-Yu Lin, Lung Chang, Wayne W. Yeh, Shih-Ching Huang, Tung-Ying Chen, Yi-Ta Hsieh, Szu-Ting Chen, Wan-Chun Li, Chin-Chen Pan, Mel Campbell, Chia-Hung Yen, Yi-Ming Arthur Chen, Pei-Ching Chang

**Affiliations:** a Institute of Microbiology and Immunology, National Yang-Ming University, Taipei, Taiwan; b Department of Pediatrics, MacKay Children’s Hospital and MacKay Memorial Hospital, Taipei, Taiwan; c Department of Medicine, MacKay Medical College, New Taipei City, Taiwan; d Department of Pathology, MacKay Memorial Hospital, Taipei, Taiwan; e Institute of Oral Biology, School of Dentistry, National Yang-Ming University, Taipei, Taiwan; f Institute of Clinical Medicine, National Yang-Ming University, Taipei, Taiwan; g Department of Dentistry, School of Dentistry, National Yang-Ming University, Taipei, Taiwan; h Cancer Progression Research Center, National Yang-Ming University, Taipei, Taiwan; i Department of Pathology, Taipei Veterans General Hospital, National Yang-Ming University, Taipei, Taiwan; j UC Davis Cancer Center, University of California, Davis, California, USA; k Center for Infectious Disease and Cancer Research, Kaohsiung Medical University, Kaohsiung, Taiwan; l Master Program in Clinical Pharmacogenomics and Pharmacoproteomics, College of Pharmacy, Taipei Medical University, Taipei, Taiwan; University of Southern California

**Keywords:** Kaposi’s sarcoma-associated herpesvirus, KSHV, human immunodeficiency virus, HIV, HIV-1 Tat, long noncoding RNA, lncRNA

## Abstract

Kaposi’s sarcoma-associated herpesvirus (KSHV) is the causative agent of Kaposi’s sarcoma (KS), an AIDS-defining cancer with abnormal angiogenesis. The high incidence of KS in human immunodeficiency virus (HIV)-infected AIDS patients has been ascribed to an interaction between HIV type 1 (HIV-1) and KSHV, focusing on secretory proteins. The HIV-1 secreted protein HIV Tat has been found to synergize with KSHV lytic proteins to induce angiogenesis. However, the impact and underlying mechanisms of HIV Tat in KSHV-infected endothelial cells undergoing viral lytic reactivation remain unclear. Here, we identified LINC00313 as a novel KSHV reactivation-activated long noncoding RNA (lncRNA) that interacts with HIV Tat. We found that LINC00313 overexpression inhibits cell migration, invasion, and tube formation, and this suppressive effect was relieved by HIV Tat. In addition, LINC00313 bound to polycomb repressive complex 2 (PRC2) complex components, and this interaction was disrupted by HIV Tat, suggesting that LINC00313 may mediate transcription repression through recruitment of PRC2 and that HIV Tat alleviates repression through disruption of this association. This notion was further supported by bioinformatics analysis of transcriptome profiles in LINC00313 overexpression combined with HIV Tat treatment. Ingenuity Pathway Analysis (IPA) showed that LINC00313 overexpression negatively regulates cell movement and migration pathways, and enrichment of these pathways was absent in the presence of HIV Tat. Collectively, our results illustrate that an angiogenic repressive lncRNA, LINC00313, which is upregulated during KSHV reactivation, interacts with HIV Tat to promote endothelial cell motility. These results demonstrate that an lncRNA serves as a novel connector in HIV-KSHV interactions.

**IMPORTANCE** KS is a prevalent tumor associated with infections with two distinct viruses, KSHV and HIV. Since KSHV and HIV infect distinct cell types, the virus-virus interaction associated with KS formation has focused on secretory factors. HIV Tat is a well-known RNA binding protein secreted by HIV. Here, we revealed LINC00313, an lncRNA upregulated during KSHV lytic reactivation, as a novel HIV Tat-interacting lncRNA that potentially mediates HIV-KSHV interactions. We found that LINC00313 can repress endothelial cell angiogenesis-related properties potentially by interacting with chromatin remodeling complex PRC2 and downregulation of cell migration-regulating genes. An interaction between HIV Tat and LINC00313 contributed to the dissociation of PRC2 from LINC00313 and the disinhibition of LINC00313-induced repression of cell motility. Given that lncRNAs are emerging as key players in tissue physiology and disease progression, including cancer, the mechanism identified in this study may help decipher the mechanisms underlying KS pathogenesis induced by HIV and KSHV coinfection.

## Abstract (precis)

KS is a prevalent tumor associated with infections with two distinct viruses, KSHV and HIV. Since KSHV and HIV infect distinct cell types, the virus-virus interaction associated with KS formation has focused on secretory factors. HIV Tat is a well-known RNA binding protein secreted by HIV. Here, we revealed LINC00313, an lncRNA upregulated during KSHV lytic reactivation, as a novel HIV Tat-interacting lncRNA that potentially mediates HIV-KSHV interactions. We found that LINC00313 can repress endothelial cell angiogenesis-related properties potentially by interacting with chromatin remodeling complex PRC2 and downregulation of cell migration-regulating genes. An interaction between HIV Tat and LINC00313 contributed to the dissociation of PRC2 from LINC00313 and the disinhibition of LINC00313-induced repression of cell motility. Given that lncRNAs are emerging as key players in tissue physiology and disease progression, including cancer, the mechanism identified in this study may help decipher the mechanisms underlying KS pathogenesis induced by HIV and KSHV coinfection.

## INTRODUCTION

Kaposi’s sarcoma (KS) is a multicentric malignancy of endothelial origin and the most common AIDS-defining neoplasm caused by Kaposi’s sarcoma-associated herpesvirus (KSHV), a gammaherpesvirus also known as human herpesvirus type 8 (HHV-8) (1). As a result of the AIDS pandemic, KS has become one of the most common cancers in developed countries, where it causes significant morbidity and mortality. However, KSHV infection is necessary but not sufficient for KS development, and other cofactors are required for malignant transformation (2, 3). Among them, human immunodeficiency virus (HIV) infection is the main cofactor, and supporting evidence includes the following: (i) the incidence of KS is 1 in 100,000 in the general population (4) but increases approximately 20,000- to 50,000-fold in HIV-infected patients (5, 6) and (ii) the highly active antiretroviral therapy (HAART) targeting HIV has markedly decreased the incidence of KS (7, 8). However, although HAART has markedly decreased the incidence of KS (9, 10), up to 50% of AIDS-associated KS (AIDS-KS) patients never achieve total remission under HAART (11). Therefore, AIDS-KS remains the second most frequent tumor in HIV-infected patients worldwide (7, 8, 12), and development of new intervention strategies is still needed.

Although it has been proposed that immunodeficiency itself is the main determinant of KS, the incidence of KS in patients with HIV infection is 300-fold higher than in persons with other types of immunosuppression (6). Moreover, individuals with KSHV-HIV coinfection have an increase of 60%/year in the probability of developing KS after HIV infection (13). Since HIV and KSHV infect different cell types (HIV infects primarily CD4^+^ T cells and tissue macrophages, and KSHV infects primarily endothelial and B cells), two crucial paracrine mechanisms have been proposed by which HIV promotes KS pathogenesis: (i) promotion of cytokine production from HIV-infected CD4^+^ T cells and (ii) secretion of HIV proteins, particularly HIV Tat (reviewed in reference 14). The detection of HIV Tat in spindle cells of AIDS-associated KS lesions (15) further supports the second notion.

Studies have initially focused on the potential role of HIV Tat protein in KS formation due to the discovery of a dermal lesion resembling KS in HIV Tat transgenic mice (16) and the promotion of endothelial cell growth (17), migration (18), angiogenesis (19), and tumorigenesis (20) by extracellular HIV Tat. The synergistic effects of HIV Tat and KSHV in modulating KS formation that have been identified include the following: (i) increasing KSHV infectivity in endothelial cells (21); (ii) increasing the expression (22), signaling activity (23, 24), and tumorigenicity (24) of vGPCR; (iii) promoting viral interleukin-6 (vIL-6)-induced angiogenesis and tumorigenesis (25); and (iv) enhancing K1-induced cell proliferation and angiogenesis (26). The acceleration of kaposin A (27)- and vIL-6 (25)-mediated tumorigenesis by HIV Tat has also been observed in fibroblasts. Interestingly, except for kaposin A, the viral genes modulated by HIV Tat are all KSHV lytic genes, suggesting that the lytic cycle of KSHV is necessary to support KS formation in HIV-infected individuals.

Mechanistic studies have revealed that HIV Tat potentiates the oncogenic activity of KSHV proteins mainly through the regulation of the PI3K/Akt pathway (23, 25, 27). In addition to signal transduction, one recent report showed that HIV Tat may synergize with a KSHV viral protein to induce the expression of microRNAs (miRNAs), a type of small noncoding RNAs (ncRNAs), which consequently leads to the induction of angiogenesis (26). Beyond miRNAs, long noncoding RNAs (lncRNAs), defined as transcripts longer than 200 nucleotides (nt) (28), are emerging as a new large class of functional ncRNAs (29). lncRNAs commonly originate from intergenic regions (known as lincRNAs) but share many features with mRNAs, including RNA polymerase II transcription, 5′-methylguanosine capping, 3′-polyadenylation, and RNA splicing (30). However, they are expressed at a lower level than mRNAs (31) and often display high tissue specificity, implying that they may function in a cell type-specific manner (29). Accumulating data suggest that lncRNAs can exhibit diverse biological functions through binding to protein, DNA, or RNA and serve as scaffolds or decoys. It is not surprising that aberrant lncRNA expression is associated with various diseases, including cancer. To date, thousands of lncRNAs have been annotated by genome-wide studies, yet the precise function has been determined for only a small population.

Recently, lncRNAs have been linked to viral infection as well (32, 33). The lncRNA uc002yug.2 enhances HIV-1 reactivation through upregulation of HIV Tat protein expression (34). The lncRNA NRON represses HIV replication through interacting with and promoting the ubiquitylation and degradation of HIV Tat (35). These findings prompted us to hypothesize that HIV Tat may potentiate KS formation through binding to lncRNAs upregulated during KSHV reactivation. To study this, we first performed a transcriptome analysis in latent and lytic KSHV-infected cells treated with or without HIV Tat and identified 60 lncRNAs that were upregulated upon KSHV reactivation in both the presence and absence of HIV Tat. RNA immunoprecipitation (RIP) and RNA pulldown assays showed that one lncRNA identified in this screen, LINC00313, interacted with HIV Tat and polycomb repressive complex 2 (PRC2). Importantly, overexpression of LINC00313 significantly inhibits the migration, invasion, and tube formation of HMEC1 cells and human umbilical vein endothelial cells (HUVECs), and this inhibition may be rescued by HIV Tat. Consistent with our cell culture data, transcriptional profiling followed by Ingenuity Pathway Analysis (IPA) showed that LINC00313 tends to inhibit cell movement and migration, and inhibition of these pathways was no longer observed after HIV Tat treatment. Together, our study revealed a novel HIV Tat-interacting lncRNA, LINC00313, that links the KSHV and HIV virus-virus interaction in mediating KS progression.

## RESULTS

### Genome-wide transcriptome profiling reveals the functional impact of HIV Tat in KSHV latent and lytic reactivated SLK cells.

**FIG 1:**
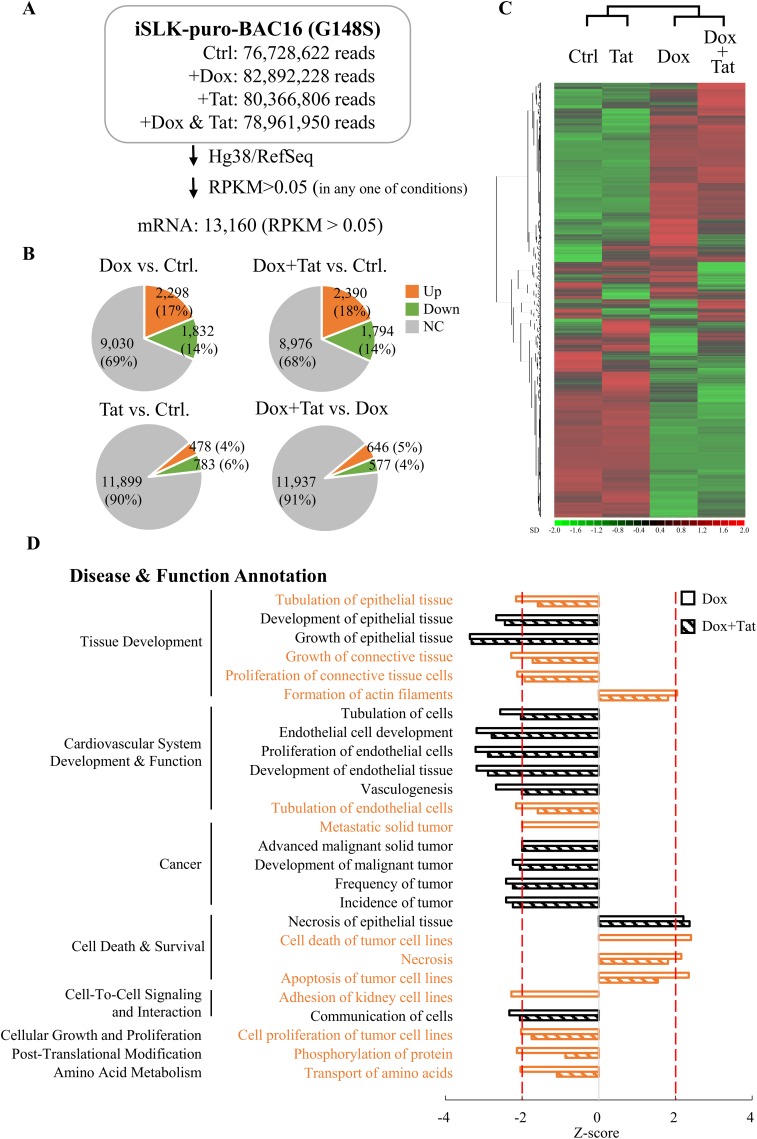
HIV Tat-regulated host genes in latent and lytic KSHV-infected SLK cells. (A) Summary of RNA-seq data. iSLK-BAC16 cells were treated with 1 μg/ml doxycycline (Dox) with or without 0.2 μg/ml HIV Tat. Cells cultured without any treatment were used as the control (Ctrl). At 48 h after treatment, total RNA was extracted and used for RNA-seq on an Illumina HiSeq 2000 platform. Paired-end reads were aligned to the human reference genome (Hg38) using CLC Genomics Workbench 11 (Qiagen) and annotated to RefSeq82 using the Partek Genomics Suite 7 (Partek). RPKM higher than 0.05 in any one of the samples were considered expressed and used for subsequent analysis. (B) Pie charts of mRNA expression data from the three treatment conditions described for panel A. The numbers (percentages) of mRNAs up- or downregulated more than 1.5-fold are shown. (C) Heat map of hierarchical cluster analysis of mRNA expression data from the 4 treatment conditions described for panel A. (D) The IPA regulation *Z*-score algorithm was applied to predict disease and function pathways that are enriched in genes differentially expressed (fold change > 1.5) in Dox- and Dox-plus-HIV Tat-treated groups. Cells without any treatment were used as the control (Ctrl). Pathways enriched in the Dox-treated group with a *Z*-score greater than 2 or less than −2 are represented by open bars. The *Z*-scores in the Dox-plus-HIV Tat treatment group were plotted in parallel (hatched bars). The pathways that showed lower significance in the Dox-plus-HIV Tat-treated group than in the Dox-treated group are shown in orange.

To explore the role of HIV Tat in KSHV-infected endothelial cells, the ability of our purified recombinant HIV Tat protein (see Fig. S1A in the supplemental material) to enter SLK cells was first examined by immunostaining. The results showed that HIV Tat was mainly localized to the nucleus (Fig. S1B), which is consistent with the detection of nuclear-localized HIV Tat after ectopic expression (Fig. S1C) and in KS clinical samples (Fig. S1D). Following this confirmation, a high-throughput RNA sequence (RNA-seq) analysis was performed using RNAs isolated from iSLK-BAC16 cells, a doxycycline (Dox)-inducible recombinant KSHV-harboring SLK cell line, that was treated with 1 μg/ml Dox in the presence or absence of 0.2 μg/ml purified HIV Tat. As shown in Fig. 1A, following alignment of the raw reads to the Hg38 human genome using CLC Genomics Workbench 11 and annotation of the transcripts with RefSeq82 using Partek Genomic Suite v.7.0 (Partek, St. Louis, MO), we identified 13,160 mRNAs (reads per kilobase of transcript per million mapped reads [RPKM] > 0.05) that were expressed in iSLK-BAC16 cells under at least one of the treatment conditions. When the gene expression profiles between latent infected and lytic reactivated iSLK-BAC16 cells that were treated with or without HIV Tat were compared, around 1,000 to 4,000 genes were regulated (≥1.5-fold change) by viral reactivation (Dox versus control, 31%) and HIV Tat treatment (HIV Tat versus control, 10%) (Fig. 1B). Unsupervised hierarchical clustering analysis showed that latent infected and lytic reactivated samples were grouped in separate clusters and that HIV Tat treatment was clustered with its corresponding untreated group, demonstrating that HIV Tat affects host gene expression in both the latent and lytic phases of the KSHV life cycle (Fig. 1C).

To explore the potential mechanisms that HIV Tat uses to modulate gene expression in SLK cells during the KSHV lytic cycle, genes that were differentially expressed upon KSHV reactivation with or without HIV Tat treatment (Fig. 1B, upper pie chart) were subjected to pathway analysis using Ingenuity Pathway Analysis (IPA) software. We used an activation *Z*-score to identify the most important downstream signaling pathways modulated by HIV Tat upon KSHV reactivation and considered a *Z*-score greater than or equal to 2 as significant activation and a *Z*-score less than or equal to −2 as significant inhibition of the functional activity identified. As shown in Fig. 1D, genes altered upon KSHV reactivation regulate 8 main functions connected to 26 cell processes. Among them, 13 pathways lost their *Z*-score significance after HIV Tat treatment (Fig. 1D). These pathways include disinhibition of cell tubulation, proliferation and metastasis, and deactivation of cell death.

### Soluble HIV Tat protein promotes cell motility and viral reactivation in KSHV lytic reactivated SLK cells.

**FIG 2:**
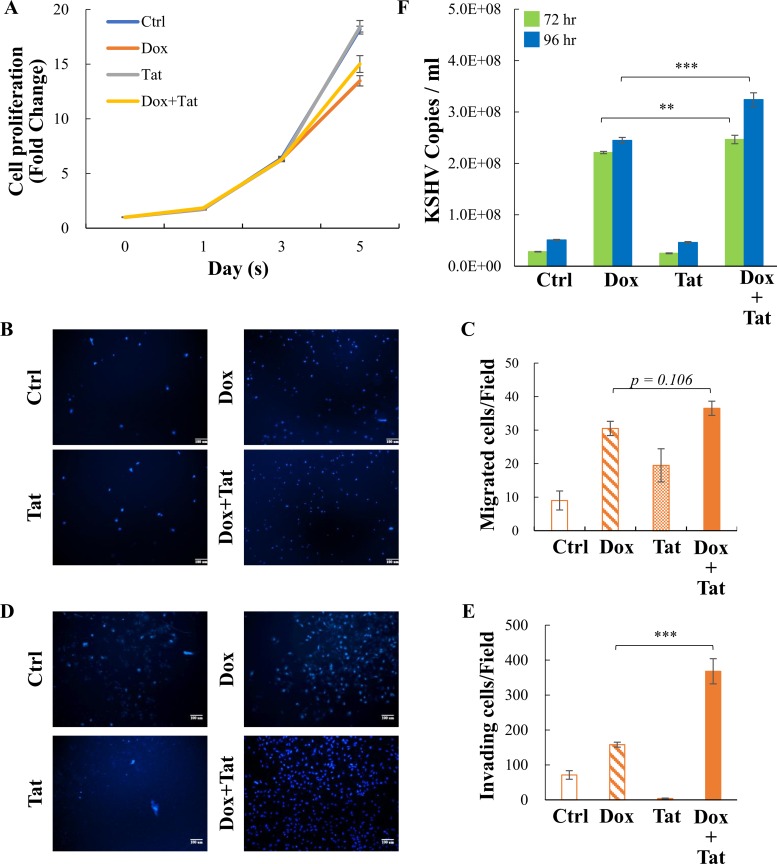
Soluble HIV Tat enhances the invasion and viral production of KSHV lytic infection-reactivated SLK cells. (A) Viability of control and iSLK-BAC16 cells treated with Dox (1 μg/ml) with or without of HIV Tat (0.2 μg/ml) at the indicated time points was assessed using MTT. (B and D) Representative images from cell migration (B) and invasion (D) assays in iSLK-BAC16 cells treated as described for panel A. (C and E) Quantification of cell migration (C) and invasion (E) described in the legend for panels B and D, respectively. (F) Supernatants from iSLK-BAC16 cells treated as described in the legend for panel A were collected at 72 and 96 h, and the viral titers were determined by analyzing the virion-associated DNA levels using TaqMan qPCR. Data shown are means ± standard deviations (SD) (*n* = 3). **, *P* < 0.01; ***, *P* < 0.005 (Student’s *t* test).

Following the pathway analysis, cell proliferation, migration, and invasion were evaluated in iSLK-BAC16 cells treated with Dox (1 μg/ml) and/or HIV Tat (0.2 μg/ml). Cells without any treatment were used as a control. Our data showed that KSHV reactivation significantly reduced cell number on day 5 compared to the control and that HIV Tat treatment might slightly recover the cell number during reactivation (Fig. 2A and Fig. S2A). This observation agrees with our IPA analysis showing that enriched cell death pathways during KSHV reactivation were diminished by HIV Tat treatment (Fig. 1D). Consistent with previous reports (18), the addition of HIV Tat had a stimulatory effect on cell migration (Fig. 2B and C). More importantly, HIV Tat slightly increased KSHV reactivation-induced cell migration (Fig. 2B and C) and significantly enhanced the properties of invasion of iSLK-BAC16 cells (Fig. 2D to E).

We further analyzed viral titers at 72 and 96 h after Dox-induced KSHV reactivation. Consistent with previous reports indicating that HIV Tat was able to enhance KSHV lytic reactivation in B lymphoma cells (36, 37), our data showed that soluble HIV Tat can also enhance the virus production in SLK cells at 96 h after KSHV reactivation but not in latent control cells (Fig. 2F; see Fig. S2B in the supplemental material). These data suggested that internalized soluble HIV Tat protein may promote angiogenesis-related functions in KSHV-infected cells through enhancement of KSHV reactivation. However, we cannot exclude the possibility that HIV Tat may enhance angiogenesis through other regulatory mechanisms, including effects on latently infected cells.

### Transcriptome profiling of lncRNAs in iSLK-BAC16 cells upon KSHV reactivation.

**FIG 3:**
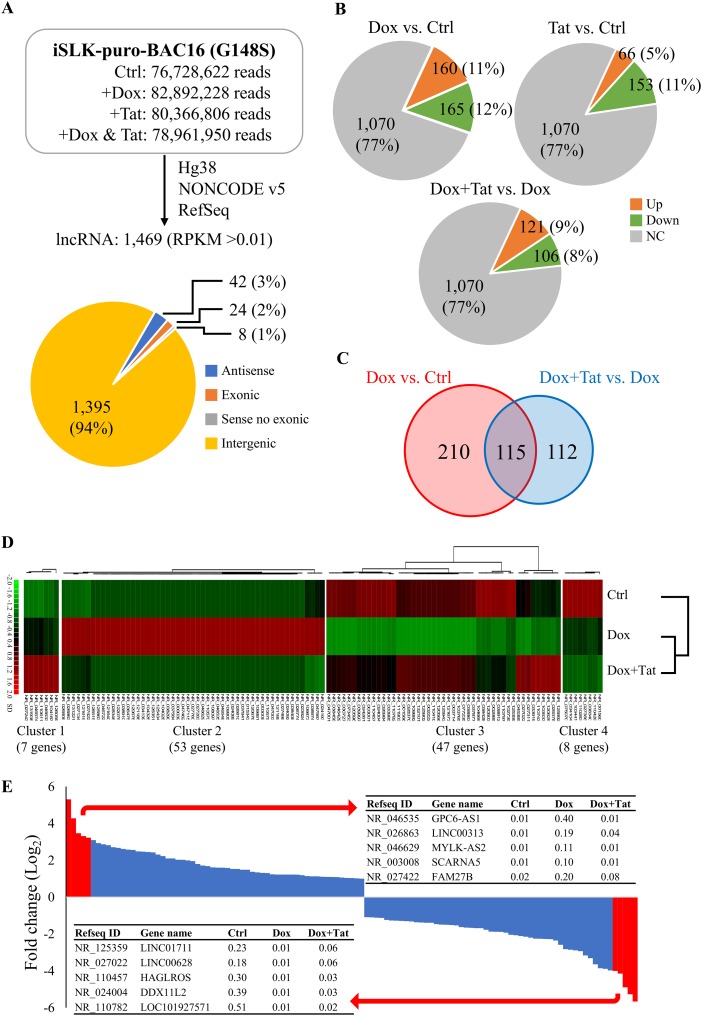
HIV Tat-regulated lncRNAs in latent and lytic KSHV-infected SLK cells. (A) Summary of lncRNA data analysis. RNA-seq data from Fig. 1 was annotated to NONCODE v5.0 using the Partek Genomics Suite 7 (Partek). RPKM higher than 0.01 in at least one sample were considered expressed and subsequently converted to RefSeq IDs. The pie chart depicts the number of lncRNAs in each category. (B) Pie charts of lncRNA expression data from the three treatment conditions described in the legend for panel A. The numbers (percentages) of lncRNAs up- or downregulated more than 2-fold are shown. (C) Venn diagram of differentially expressed lncRNAs identified by Dox treatment (red) and by Dox-plus-HIV Tat treatment (blue), with Dox-treated cells as the control (Ctrl). (D) Heat map of hierarchical cluster analysis of the 115 lncRNAs shown in panel C that were differentially expressed under both Dox versus Ctrl and Dox-plus-HIV Tat versus Dox conditions. (E) Histogram of the fold change (log_2_) from Dox versus Ctrl of the 115 lncRNAs shown in panel C that were differentially expressed in both Dox versus Ctrl and Dox-plus-HIV Tat versus Dox conditions. The top 5 lncRNAs that were up- and downregulated by Dox treatment are listed according to their RPKM.

**FIG 4:**
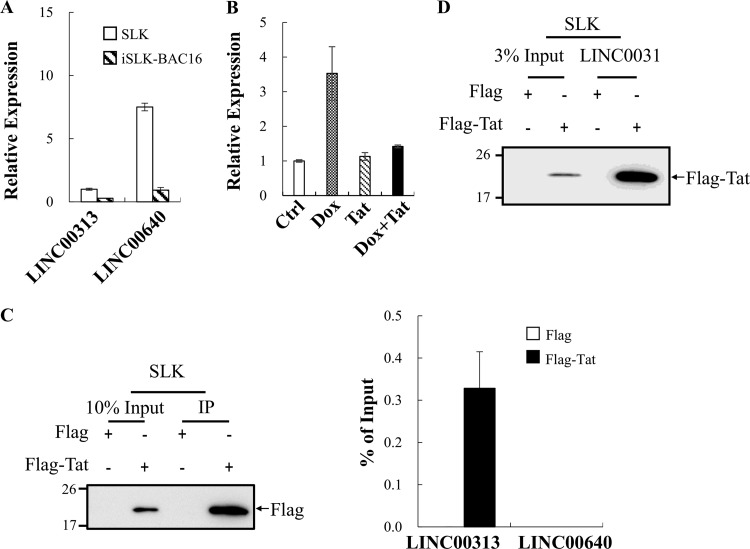
LINC00313 interacts with HIV Tat. (A) Expression levels of LINC00313 and LINC00640 in SLK and iSLK-BAC16 cells were determined by RT-qPCR. Values are relative to the level of LINC00313 observed in SLK cells. (B) Relative expression levels of LINC00313 in control and iSLK-BAC16 cells treated with Dox (1 μg/ml) with or without HIV Tat (0.2 μg/ml) for 48 h was assessed by RT-qPCR. (C) RNA immunoprecipitation (RIP) was perform in pcDNA3-Flag- and pcDNA3-Flag-HIV Tat-transfected SLK cells (1 × 10^7^ cells per IP) using anti-Flag antibody. Successful immunoprecipitation of Flag-HIV Tat was verified by immunoblotting using anti-Flag antibody (left panel). HIV Tat-associated RNA was analyzed by RT-qPCR (right panel). (D) RNA pulldown assay was performed by incubation of *in vitro*-transcribed biotin-labeled LINC00313 with total cell lysates (TCLs) prepared from SLK cells transfected as described for panel C. Products pulled down by streptavidin beads were examined by immunoblotting using anti-Flag antibody.

In recent years, thousands of lncRNAs have been identified by high-throughput sequence technology. Increasing numbers of reports suggest that lncRNAs participate in multiple cellular processes. In an attempt to identify lncRNAs whose expression is differentially regulated in cells upon KSHV reactivation and HIV Tat treatment, we reannotated the RNA-seq reads with NONCODE v5.0 using Partek Genomic Suite v.7.0 (Partek, St. Louis, MO), followed by changing the NONCODE designations (IDs) to RefSeq IDs. We identified 1,469 lncRNAs (RPKM > 0.01) that were expressed in iSLK-BAC16 cells under at least one treatment condition (Fig. 3A). Among them, 325, 219, and 227 lncRNAs were differentially regulated (≥2-fold change) by Dox (23%), HIV Tat (16%), and Dox-plus-HIV Tat treatment, respectively (Fig. 3B). To identify lncRNAs that were regulated by HIV Tat under KSHV reactivation conditions, the lncRNAs that were differentially regulated under Dox treatment in the absence (325) and presence (227) of HIV Tat were compared, and 115 lncRNAs were identified (Fig. 3C). Unsupervised hierarchical clustering analysis grouped these lncRNAs into 4 distinct clusters (Fig. 3D). The Dox-upregulated lncRNAs that were upregulated (7) or downregulated (38) by HIV Tat under KSHV reactivation conditions were grouped into clusters 1 and 2, respectively. Clusters 3 and 4 contained Dox-downregulated lncRNAs that were upregulated (39) or downregulated (8) by HIV Tat treatment upon KSHV reactivation, respectively. As shown in Fig. 3E, the 115 lncRNAs were plotted by fold change (log_2_) in expression upon KSHV reactivation, and the top 5 up- and downregulated lncRNAs were listed. However, using reverse transcriptase quantitative PCR (RT-qPCR) analysis, we could consistently detect only LINC00313 among the top 5 KSHV reactivation-upregulated lncRNAs in SLK and iSLK-BAC16 cells (Fig. 4A and Fig. S3); we therefore focused our efforts on this lncRNA.

### LINC00313 is upregulated during KSHV reactivation and interacts with HIV Tat.

Since lncRNAs can bind to various proteins to form functional complexes and HIV Tat is a well-known RNA-binding protein, we further asked whether HIV Tat may modulate cellular function by interacting with KSHV reactivation-upregulated lncRNAs. To this end, an RNA immunoprecipitation (RIP) experiment was performed. Following confirmation of LINC00313 upregulation upon KSHV reactivation (Fig. 4B), RIP was conducted in SLK cells overexpressing Flag-tagged HIV Tat. lncRNA LINC00640 that was expressed at a relative high level in SLK cells was included as a control (Fig. 4A). Following RIP, immunoblotting was first performed to confirm the successful immunoprecipitation of HIV Tat (Fig. 4C, left panel). RIP followed by RT-qPCR showed the enrichment of LINC00313 by Flag-HIV Tat but not the Flag-tagged control (Fig. 4C, right panel). These data indicate that HIV Tat may potently bind to endogenous LINC00313 in SLK cells. This interaction was further confirmed by an *in vitro* RNA pulldown assay. Total cell lysates (TCLs) prepared from SLK cells overexpressing the Flag tag or Flag-tagged HIV Tat were incubated with biotinylated LINC00313 RNA and subjected to the pulldown procedure using streptavidin beads. Immunoblotting results confirmed the interaction between HIV Tat and LINC00313 (Fig. 4D).

### Exogenous LINC00313 inhibits endothelial cell motility and tube formation.

**FIG 5:**
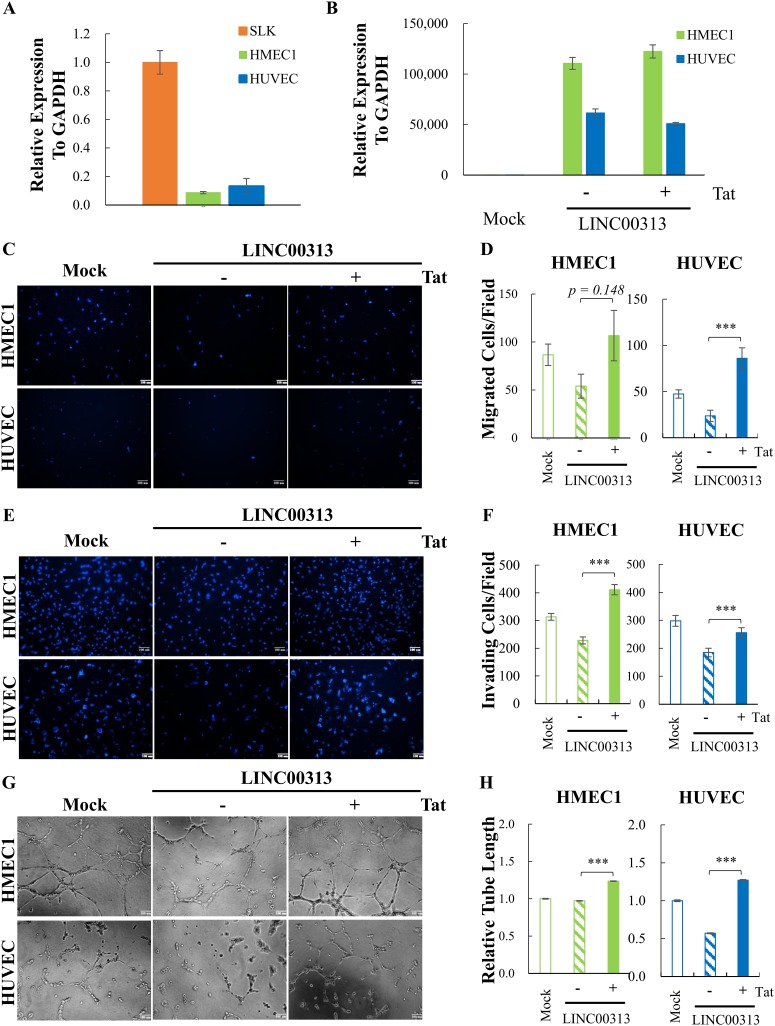
HIV Tat reverses the inhibition of motility, invasion, and tube formation by LINC00313. (A) The level of LINC00313 in HMEC1, HUVEC, and SLK cells was determined by RT-qPCR. (B) HMEC1 and HUVEC were transduced with control or lentivirus-expressing LINC00313. At 24 h after transduction, cells were treated with or without HIV Tat for another 48 h. Successful overexpression of LINC00313 in HMEC1 and HUVEC was determined by RT-qPCR. (C, E, and G) Representative images from cell migration (C), invasion (E), and tube formation (G) assays of HMEC1 cells (upper panel) and HUVECs (lower panel) treated as described for panel B. (D, F, and H) Quantification of cell migration (D), invasion (F), and tube formation (H) of HMEC1 cells (left panel) and HUVECs (right panel) as described for panels C, E, and G, respectively. Data shown are means ± SD (*n* = 3). ***, *P* < 0.005 (Student’s *t* test).

To probe the angiogenic role of LINC00313 by using an overexpression strategy, the expression level of LINC00313 was first analyzed in human endothelial cell lines HMEC1 and HUVEC, together with SLK cells. RT-qPCR data showed that HMEC1 cells and HUVECs express relatively low levels of LINC00313 (Fig. 5A), and they were used for ectopic expression experiments. Following transduction of HMEC1 cells and HUVECs with control or lentivirus-expressing LINC00313, the successful overexpression of LINC00313 was first confirmed by RT-qPCR (Fig. 5B). The LINC00313-overexpressed HMEC1 cells and HUVECs treated with or without HIV Tat were subjected to migration, invasion, and tube formation assays. Cells transduced with mock lentivirus were used as a control. Surprisingly, overexpression of LINC00313 inhibited cell migration (Fig. 5C and D), invasion (Fig. 5E and F), and tube formation (Fig. 5G and H) in both HMEC1 cells and HUVECs. Most importantly, the HIV Tat treatment antagonized the inhibition induced by LINC00313 overexpression. These results suggest that HIV Tat may disengage LINC00313 from its original regulatory context.

### HIV Tat antagonized LINC00313 function through inhibition of the LINC00313-PRC2 interaction.

**FIG 6:**
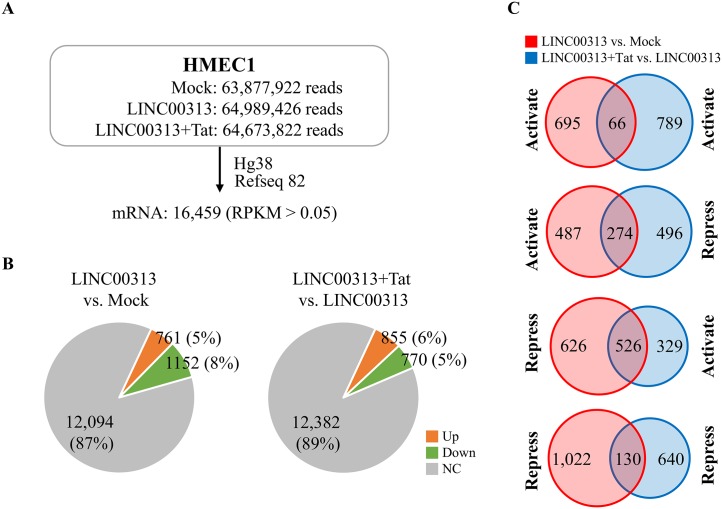
LINC00313-regulated cell movement-related genes in HMEC1 cells. (A) Summary of RNA-seq data. Total RNA extracted from HMEC1 cells treated as described in the legend to Fig. 5B were subjected to RNA-seq. Paired-end reads were aligned and annotated as described in the legend to Fig. 1A. RPKM higher than 0.05 in at least one sample were considered expressed and used for subsequent analysis. (B) Pie chart of mRNA expression data from LINC00313-overexpressing HMEC1 cells treated as described for panel A. The numbers (percentages) of mRNAs up- or downregulated more than 1.5-fold are shown. (C) Venn diagrams of differentially expressed mRNAs identified in LINC00313-overexpressing treatment (red) and in LINC00313-overexpressing-plus-HIV Tat treatment using overexpression cells as the Ctrl (blue).

To elucidate the mechanisms responsible for LINC00313 that were opposed by HIV Tat, the genes regulated by LINC00313 overexpression in the presence or absence of HIV Tat were revealed by an RNA-seq survey of HMEC1 cells (Fig. 6A). The analysis indicated that more genes were downregulated (1,152; 8%) by LINC00313 overexpression than were upregulated (761; 5%) in comparison with the control (Fig. 6B, left pie chart). Interestingly, following HIV Tat treatment, more genes were upregulated (855; 6%) than downregulated (770; 5%) (Fig. 6B, right pie chart). With additional dissection of LINC00313-downregulated genes, we found that 526 (45.6%) of the genes became upregulated upon HIV Tat treatment and only 130 (11.2%) of the genes remained downregulated (Fig. 6C). Bioinformatics analysis of LINC00313-downregulated genes (1,152) using IPA showed a total of 54 pathways (Table S1) that were significantly enriched with *Z*-score hits of ≥2.0. Among them, only 4 pathways (Table S1) were identified after HIV Tat treatment. This result suggests that HIV Tat may relieve LINC00313-mediated transcription repression.

**FIG 7:**
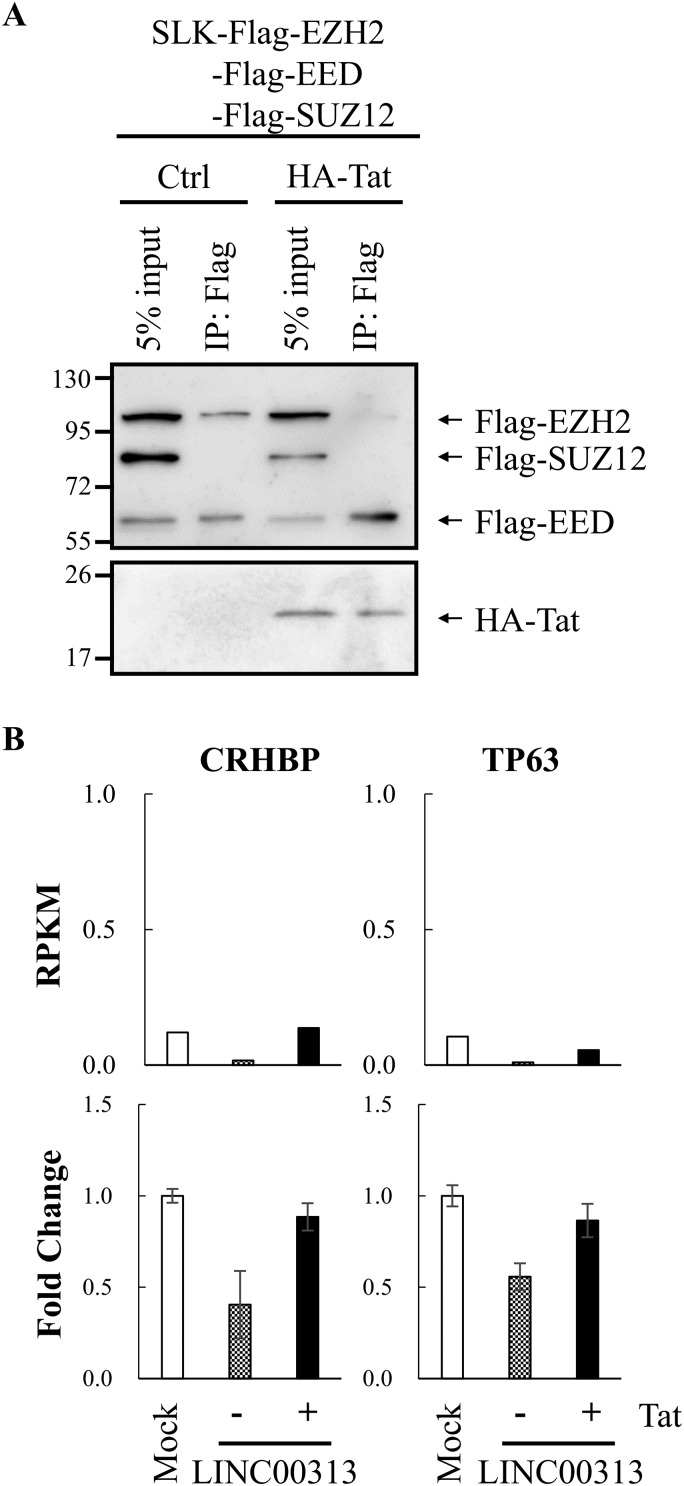
LINC00313 interacted with EZH2, and HIV Tat reduced this association. (A) An RNA pulldown assay was performed by incubation of *in vitro*-transcribed biotin-labeled LINC00313 with TCLs prepared from SLK cells transfected with pcDNA3-Flag-EED, pcDNA3-Flag-EZH2, and pcDNA3-Flag-SUZ12 with or without pcDNA3-HA-HIV Tat. Products pulled down by streptavidin beads were examined by immunoblotting using anti-Flag and anti-HA antibodies. (B) Expression levels of CRHBP and TP63 in HMEC1 cells treated as described in the legend to Fig. 5B were determined by RT-qPCR.

Among chromatin remodeling complexes shown to be recruited by lncRNAs, PRC2, a histone H3 lysine 27 (H3K27) methyltransferase complex, is one of the most well studied (40). Since H3K27me3 is a well-known repressive histone mark (41), we hypothesized that LINC00313 may be a novel PRC2-interacting lncRNA. HIV Tat may interact with LINC00313 and disrupt this association. To study this, we performed an RNA pulldown experiment. TCLs prepared from SLK cells overexpressing Flag-tagged EED, EZH2, and SUZ12 alone or with hemagglutinin (HA)-tagged HIV Tat were incubated with biotinylated *in vitro*-transcribed LINC00313 RNA and subjected to the pulldown procedure using streptavidin beads. Consistent with our hypothesis, immunoblotting results showed that LINC00313 interacts with components of PRC2 (Fig. 7A). Interestingly, the interaction between LINC00313 and EZH2 was inhibited by HIV Tat overexpression (Fig. 7A). These data suggest that LINC00313 may mediate transcription repression through recruitment of the PRC2 complex. HIV Tat may interact with LINC00313 and disrupt this association. This notion was further supported by RT-qPCR analysis of two LINC00313-repressed genes, those encoding corticotropin releasing hormone binding protein (CRHBP) and tumor suppressor protein p63 (TP63) (Fig. 7B), whose expression patterns confirmed Tat-dependent derepression of LINC00313-mediated transcriptional effects.

## DISCUSSION

Preexisting HIV infection followed by secondary KSHV infection induces KS development in up to 50% of HIV-positive patients, demonstrating that KSHV is one of the most potent oncogenic human viruses in this special setting (42). However, KSHV infection alone is not sufficient to induce KS development. This suggests that virus-virus interactions between KSHV and HIV are required for malignant transformation. Since the KS lesion is of endothelial cell origin and HIV does not directly infect endothelial cells, studies have focused on elucidating the circulating soluble HIV proteins that may potentiate neoplastic processes (24 – 27, 43 – 45). Previous reports indicated that viral proteins secreted by HIV-infected cells may be taken up by KSHV-infected cells and consequently dysregulate the KSHV latent to lytic infection switch (39, 46) and promote disease progression (24 – 27, 43, 45). However, it should be note that KS tumor cells are almost exclusively latently infected (1 – 5). Thus, the effects of HIV secretory proteins may be limited to early stages of KS pathogenesis, when lytic replication can promote KS, or to a subpopulation of infected cells undergoing lytic replication against a larger background of latently infected tumor cells.

It has long been known that HIV Tat promotes a KS-related phenotype, including migration, angiogenesis, and tumorigenesis (18 – 20). Studies focused on the interaction between KSHV and HIV Tat showed that the synergistic effect of HIV Tat and KSHV in KS formation can be explained, in part, by an increase in KSHV infectivity of endothelial cells (21). In addition, both intracellular, overexpressed HIV Tat protein and extracellular, treated HIV Tat protein have been shown to enhance KSHV lytic reactivation in B lymphoma cells (36, 37). While HIV load may correlate with the production of HIV Tat, HIV infection has also been identified as responsible for inducing KSHV reactivation (47 – 49). Since persistent lytic reactivation of KSHV is a prerequisite for KS pathogenesis, these results implicate the induction of KSHV reactivation by HIV Tat as a participant in promoting KS pathogenesis (21, 50). In line with this, accumulating reports have shown that both soluble HIV Tat and ectopically expressed HIV Tat are able to synergize KSHV latent and lytic viral protein-mediated angiogenesis and tumorigenesis (22, 24, 25). However, most of the KSHV reactivation studies have been done in primary effusion lymphoma (PEL) cells, not in endothelial cells, the cell type of KS tumor origin. Moreover, the knowledge gained has been largely dependent on overexpression of individual KSHV proteins and not on the whole viral context. Here, by using virus-containing SLK cells, we confirmed that KSHV reactivation increased cell migration and invasion ability and that these phenotypes can be further enhanced by HIV Tat (Fig. 2B to E). Our data showed that HIV Tat was also able to enhance KSHV reactivation in SLK cells after prolonged treatment (Fig. 2F). Surprisingly, bioinformatics analysis of transcriptome data from KSHV-containing SLK cells undergoing lytic reactivation showed inhibition of tubulation and metastasis (*Z*-score less than or equal to −2) (Fig. 1D). This result suggests that though many KSHV lytic proteins have angiogenesis potential, in the whole virus context, KSHV reactivation tends to modulate genes negatively associated with cell migration and tube formation. This may also explain why KSHV infection alone induces KS inefficiently. The loss of tubulation and metastasis pathways after HIV Tat treatment (Fig. 1D) suggests that HIV Tat may enhance an angiogenesis phenotype through interference with those pathways. Although our initial KSHV-related studies were carried out in SLK cells of epithelial origin, we subsequently confirmed these findings using endothelial cell lines HMEC1 and HUVEC (Fig. 5 and 6).

It should be noted that HIV Tat protein has been detected in the serum of HIV-infected patients at levels in the nanomolar range (38, 51). The levels of HIV Tat protein required for increased KSHV infectivity (10 nM) were 10- to 15-fold higher than the HIV Tat levels detected in serum collected from HIV-infected patients (21). Moreover, soluble HIV Tat protein used to study its synergistic effect with KSHV Orf-K1 protein was 0.2 μg/ml (13 nM) (26). It is likely that the concentration of HIV Tat in KS is underestimated, since the amount of Tat protein may be much higher in the vicinity of HIV-infected cells (52). Moreover, the proportion of Tat protein might be concentrated on the surface of the cell membrane via adsorption by heparan sulfates (53, 54). Following our immunofluorescence assay (IFA) result showing that HIV Tat can be efficiently internalized into SLK cells after incubation of cells with 0.2 to 1 μg/ml (13 to 65 nM) of HIV Tat, 0.2 μg/ml of HIV Tat was used in this study.

Interestingly, previous studies have shown that miRNAs play important roles in mediating the synergistic effect of HIV auxiliary proteins in KSHV-mediated angiogenesis in tumor progression (26, 43, 45). Emerging evidence shows that in addition to small noncoding RNAs such as miRNAs, mammalian cells also have lncRNAs with multiple biological functions. lncRNAs have been shown to regulate gene expression through direct interaction with other biomolecules, such as proteins and miRNAs, and function either as scaffolds to recruit proteins to or as sponges to remove miRNAs and proteins from their targets. Interestingly, HIV Tat is a well-known RNA binding protein and has recently been found to interact with lncRNA NRON (35). Here, we identified KSHV reactivation-upregulated LINC00313 (Fig. 3D) as a novel HIV Tat-interacting lncRNA (Fig. 4). lncRNAs have been long proposed to function as epigenetic regulators that recruit histone-modifying complexes, such as PRC2 (40), and consequently repress gene expression. Transcriptome analysis revealing more genes to be downregulated than upregulated by LINC00313 overexpression (Fig. 6B) supported the notion that LINC00313 may also function as a repressive epigenetic regulator. More importantly, ectopic expression of LINC00313 inhibited genes involved in cell movement and migration pathways (Fig. 6), as well as the migration and tube formation ability of endothelial cells (Fig. 5), and this inhibition can be rescued by treatment with HIV Tat (Fig. 5 and 6). Taken together, these data suggest that HIV Tat may interact with LINC00313 and consequently inhibit its function. However, since our RT-qPCR data showed that the LINC00313 level was significantly reduced upon HIV Tat treatment (Fig. 4A), we cannot exclude the possibility that HIV Tat may also modulate the transcription and/or stability of LINC00313. Our data here provide the first evidence that HIV Tat may also be capable of inducing angiogenesis and tumorigenesis in KSHV-infected cells by targeting KSHV reactivation-upregulated lncRNAs.

In summary, our work revealed LINC00313 as an lncRNA that is upregulated during KSHV reactivation, which inhibits endothelial cell migration and tube formation. Our biological studies together with bioinformatics analysis suggest that HIV Tat can interact with LINC00313 and rescue the endothelial cell migration and tube formation ability inhibited by LINC00313. These results not only provide a potential mechanism by which HIV Tat enhances endothelial cell angiogenesis function under KSHV reactivation conditions but also suggest that the lncRNA-HIV Tat axis may be responsible for the HIV-KSHV interaction that contributes to KS progression. Further studies will be needed to understand how LINC00313 is upregulated upon KSHV reactivation and whether other KSHV-regulated lncRNAs are also involved in HIV Tat-mediated KS pathogenesis.

## MATERIALS AND METHODS

### Cells, transfection, and transduction.

SLK cells were submitted for DNA short-tandem-repeat (STR) profiling of eight highly polymorphic loci of STRs plus amelogenin (AMEL) and confirmed that these cells are most closely related to the Caki-1 cell line of epithelial origin (55). 293T, SLK, and iSLK-BAC16 cells were cultured in Dulbecco’s modified Eagle’s medium (DMEM) (Gibco; catalog no. 12100-038) containing 10% fetal bovine serum (FBS) (Gibco; 26140-079), 1% penicillin-streptomycin (Gibco; 15140-122), and 1% l-glutamine (Sigma; G8540-100G). iSLK-BAC16 cells were further supplemented with 1 μg/ml puromycin (Sigma; P8833), 250 μg/ml G418 (Sigma; A1720), and 300 μg/ml hygromycin B (Invitrogen; 106871010). For K-Rta induction, iSLK-BAC16 cells were treated with 1 μg/ml of doxycycline (Dox). HMEC1 (human dermal microvascular endothelial) cells were cultured in MCDB131 medium (Gibco; 2027-357) supplemented with 10% FBS, 20 ng/ml epidermal growth factor (EGF) (PRO-SPEC; cyt-217), 5 μg/ml hydrocortisone, and 1% l-glutamine. HUVECs (human umbilical vein endothelial cells) were cultured in EGM2 (Lonza; CC3156) with supplements (Lonza; CC4176). All cells were maintained at 37°C in a 5% CO_2_ incubator. Insect Sf9 cells were maintained in Grace’s insect medium (Gibco; 11605-094) supplemented with 10% FBS, 1% gentamicin (Gibco; 15710-064), 1% poloxamer 188 solution (Sigma; P5556), and 1% l-glutamine at 27°C.

For transfection, SLK cells were seeded at 2 × 10^5^ cells/well in a 6-well plate. Two micrograms of DNA was transfected in each well using X-tremeGENE HD DNA transfection reagent (Roche; catalog no. 06366236001) according to the manufacturer’s instructions. For transduction, the control or pLenti4-CMV/TO-LINC00313 plasmid (0.4 μg) was cotransfected with packaging plasmids pCMV-ΔR8.91 (0.3 μg) and pCMV-VSV-G (0.1 μg) into 293T cells by TransFectin lipid reagent (Bio-Rad; 170-3351). At 72 h after transfection, supernatants were collected, filtered, mixed with Polybrene at a final concentration of 8 μg/ml, and applied to HMEC1 cells or HUVECs. Infection was accomplished by spin inoculation at 1,000 × *g* for 2 h at room temperature (RT), followed by incubation at 37°C in a 5% CO_2_ incubator for another 2 h.

### Plasmids.

HIV-1 Tat cDNA (306 bp) was obtained from pLEX-Flag-Tat plasmid (provided by Yi-Ming Arthur Chen, Taipei Medical University) by PCR amplification using primers 5′-AAACGGTCCGGCCACCATGGAGCCA-3′ and 5′-AAACGGACCGCTAATCGACCGGATCTGTC-3′. The amplified PCR products were digested with RsrII (CpoI) and cloned into pFastBAC1-Flag vector. Human LINC00313 cDNA was amplified by reverse transcriptase PCR (RT-PCR) using primers 5′-AAACGGTCCGCTAGAGAGCTGGCGCACCTCCTGGGCCTGA-3′ and 5′-AAACGGACCGGCGGCCGCTCCTCATGCGTCAGGGGCCTCTTT-3′. The amplified PCR products were digested with RsrII (CpoI) and cloned into pcDNA3-Flag and pLenti4-CMV/TO plasmids.

### Construction of recombinant baculovirus and purification of HIV Tat.

pFastBAC1-Flag-HIV Tat was used to introduce the HIV Tat gene into a baculovirus shuttle vector (bacmid) propagated in DH10Bac competent cells (Invitrogen; catalog no. 10361-012) by using the Bac-to-Bac baculovirus expression system according to the manufacturer’s instructions. The bacmid was purified by using a Qiagen plasmid purification minikit (Qiagen; 12123) according to the manufacturer’s instructions. Recombinant baculovirus expressing soluble HIV Tat was generated by transfection of the purified bacmid into Sf9 cells by using X-tremeGENE HD DNA transfection reagent. At 72 h after transfection, the viruses were collected. After subsequent passages, high-titer baculovirus was generated and used for overexpression of HIV Tat in Sf9 cells.

For protein purification, Sf9 cells were infected with baculovirus expressing HIV Tat. At 72 h postinfection, cells were harvested, washed with ice-cold phosphate-buffered saline (PBS), resuspended in 10 packed-cell volumes of ice-cold hypotonic buffer (10 mM HEPES [pH 7.9], 10 mM KCl, 1 mM phenylmethylsulfonyl fluoride [PMSF], and 1× protease inhibitor [Roche, 04693132001]) containing 0.5% NP-40, and swollen on ice for 15 min. The cell lysate was clarified by centrifugation (10,000 × *g* for 10 min at 4°C) and incubated with anti-Flag M2 magnetic beads (Sigma; M8823) for 3 h at 4°C on a rotating wheel (40 to 50 rpm). The beads were washed 3 times with 500 μl of hypotonic buffer, and HIV Tat protein was eluted by adding 3× Flag peptide (Sigma; F4799) at a final concentration of 150 μg/ml for 30 min at RT. Eluted HIV Tat protein was concentrated by using Amicon Ultra 0.5-ml centrifugal filters (3-kDa molecular weight cutoff [MWCO]) (Amicon; UFC500324) and analyzed by 15% SDS-PAGE, followed by Coomassie blue staining.

### IFA.

For the immunofluorescence assay (IFA), SLK cells were seeded on coverslips (Marienfeld; catalog no. 0111550) and transfected with pcDNA3-Flag-Tat or treated with purified HIV Tat. At 48 h after transfection or incubation, cells were fixed with 4% paraformaldehyde (Alfa Aesar; 43368) in PBS for 20 min at RT, permeabilized with 0.5% Triton X-100 for 15 min at RT, blocked with 1% bovine serum albumin (BSA) in PBS for 1 h at RT, and stained overnight at 4°C with anti-Flag antibody (Sigma; F3165), followed by 1 h of incubation at RT with anti-mouse IgG(H&L) and Alexa Fluor 488 (Jackson ImmunoResearch; 115-545-003). PBS washes were performed between each step. After the final wash, cell nuclei were counterstained with Hoechst 33342 (Invitrogen; H3569) in PBS for 5 min at RT, mounted with mounting solution (Dako; S3023), and photographed using a fluorescence microscope (Leica; DMI4000B).

### IHC.

Paraffin-embedded KS specimens from HIV-negative and high-viral-load HIV-positive patients were collected at MacKay Memorial Hospital. The ethics of the study was approved by the Institutional Review Board of National Yang-Ming University (YM107135E) and MacKay Memorial Hospital (18MMHIS050e). Tissue sections were stained by immunohistochemistry (IHC) as described in our previous report (56). Anti-HIV Tat antibody (Abcam; ab43014) was used at a 1:100 dilution. The histology was evaluated by Tung-Ying Chen and validated by a board-certified pathologist, Chin-Chen Pan, in a blind fashion.

### RNA-seq and data analysis.

Total RNA was prepared from iSLK-BAC16 cells treated with and without Dox (1 μg/ml) and soluble HIV Tat (0.2 μg/ml) and from HMEC1 cells transduced with or without LINC00313 in the presence and absence of HIV Tat (0.2 μg/ml) using TRIzol (Invitrogen; catalog no. 15596018). Paired-end 100-bp high-throughput RNA sequencing (RNA-seq) was performed by the sequencing core facility of the cancer progression research center at National Yang-Ming University using Illumina HiSeq 2000. The raw reads were aligned to human reference genome GRCh38/Hg38. Transcript levels were expressed as reads per kilobase of transcript per million mapped reads (RPKM) with mRNA and lncRNA information obtained from RefSeq82 and NONCODE v5.0, respectively, using the Partek Genomics Suite. The lncRNAs IDs from NONCODE v5.0 were converted into RefSeq IDs using NONCODE ID conversion (http://www.noncode.org/id_conversion.php). Differential expression of mRNAs and lncRNAs was analyzed by comparing RPKM and calculated as fold change. To determine the expression profile similarity between KSHV reactivation and HIV Tat treatment, iSLK-BAC16 cells treated with and without Dox (1 μg/ml) and soluble HIV Tat (0.2 μg/ml) were analyzed by hierarchical clustering using dChip software. The differential expressed mRNAs were subjected to disease and function analysis using Ingenuity Pathway Analysis software (Qiagen).

### MTT assay.

A total of 1 × 10^3^ iSLK-BAC16 cells were seeded on 96-well plates and treated with Dox (1 μg/ml) in the presence or absence of HIV Tat. The cell number was measured by adding MTT [3-(4,5-dimethyl-2-thiazolyl)-2,5-diphenyl-2H-tetrazolium bromide] (Sigma; M5655) solution to each well at a final concentration of 0.5 ng/μl for 4 h at 37°C, followed by the solubilization of formazan crystals by adding an equal volume of acidified detergent (10% SDS in 0.01 M HCl). The absorbance was measured spectrophotometrically at 570 and 660 nm with a microplate reader (Synergy HTX; Bio-Tek). The culture medium was used as a blank control.

### Migration, invasion, and tube formation assay.

The iSLK-BAC16 cells were treated with Dox (1 μg/ml) with or without HIV Tat (0.2 μg/ml) for 48 h. LINC00313-overexpressed HMEC1 cells and HUVECs were cultured in the absence or presence of HIV Tat (0.2 μg/ml) for 48 h. Cells were then trypsinized, counted using trypan blue, and plated for each assay. For migration assays, cells were seeded in a 24-well transwell insert (8-μm pore size) (Costar; 3422) at a density of 1 × 10^4^ (iSLK-BAC16 and HMEC1) and 2 × 10^4^ (HUVEC) cells/well and incubated for 6 h at 37°C. For invasion assays, 1 × 10^5^ (HMEC1 and HUVEC) cells were seeded in a 24-well transwell insert (8-μm pore size) (Costar; 3422) coated with 4-fold-diluted Cultrex basement membrane extract (BME; 100 μl/well) (Trevigen; 3433-005-01) and incubated for 6 h at 37°C. Following incubation, cells were washed, fixed with 4% paraformaldehyde, and stained with Hoechst 33342 in PBS. The migrated cells were captured by fluorescence microscopy in 3 to 5 randomly chosen fields (magnification, ×100) and quantified by Metamorph (Molecular Devices). For tube formation, 2 × 10^4^ (HMEC1 and HUVEC) cells were seeded in 96-well plates coated with Cultrex BME (50 μl/well) for 6 h at 37°C. Following incubation, cells were photographed using an inverted light microscope (Leica; DMI4000B) in 5 to 10 randomly chosen fields (magnification, ×100), and the total tube length was measured using ImageJ (FIJI) software.

### Assays of KSHV viral titer.

To assess viral titers, supernatants from 1 × 10^5^ control and iSLK-BAC16 cells treated with Dox (1 μg/ml) with or without HIV Tat (0.2 μg/ml) were collected after 72 or 96 h of treatment. The KSHV viral DNA was isolated using a QIAamp MinElute virus kit (Qiagen; catalog no. 57704) according to the manufacturer’s instructions. KSHV copy numbers were determined by quantitative TaqMan PCR (qPCR) using primers (5′-CGAATACCGCTATGTACTCAGA-3′ and 5′-CGCCTCATACGAACTCCAG-3′) and a probe (5′-/56-FAM/TCAGAACATCACC ACCCCACAGAC/3BHQ_1/-3′) designed from KSHV Orf73. Real-time qPCR was performed by using iTaq universal probes supermix (Bio-Rad; 172-5132) in a CFX Connect real-time PCR detection system (Bio-Rad). Totals of 1 × 10^7^, 1 × 10^5^, 1 × 10^3^, and 1 × 10^1^ copies/μl of pcDNA3-Orf73 plasmid were used for standardization, and the actual number of virion particles was quantified by use of a standard curve.

### RNA extraction and real-time RT-qPCR.

Total RNA was extracted by TRIzol (Invitrogen; catalog no. 15596018) according to the manufacturer’s instructions. The cDNA was synthesized by reverse transcription of 1 μg RNA using random hexamers and a Maxima first-strand cDNA synthesis kit (Thermo Scientific; K1641) according to the manufacturer’s instructions. qPCR primer pairs were designed by PerlPrimer (http://perlprimer.sourceforge.net/) software. qPCR primer sequences used in this study were as follows: for LINC00313, 5′-CCTGGATTGCATAAAGGCTC-3′ and 5′-GGATCTGCAGAAACACTCTC-3′; for LINC00640, 5′-GTCCTTACTCCCAGTCTTTCC-3′ and 5′-AGCAAGACCCACGTTCTC-3′; and for GAPDH, 5′-TATGATGACATCAAGAAGGTGG-3′ and 5′-GTTGTCATACCAGGAAATGAG-3′. qPCR was performed with SYBR green master mix (Bio-Rad; 170-882Ap) on a CFX Connect real-time PCR detection system (Bio-Rad).

### RIP assay.

RNA immunoprecipitation (RIP) was performed using a Magna RIP RNA-binding protein immunoprecipitation kit (Millipore; catalog no. CR203146) by following the manufacturer’s protocol. Briefly, SLK cells were transfected with pcDNA3-Flag or pcDNA3-Flag-Tat for 48 h and used for RIP experiments with the anti-Flag M2 magnetic beads. The resultant immunoprecipitants and 10% total cell lysates (TCLs) were subjected to immunoblotting using anti-Flag antibody. The coprecipitated RNAs were extracted by acid phenol-chloroform, pH 4.5 (Ambion; AM9722). The levels of LINC00313 and LINC00640 were examined by real-time RT-qPCR.

### *In vitro* transcription and RNA pulldown assay.

To synthesize biotinylated LINC00313 RNA by *in vitro* transcription using a MEGAscript T7 transcription kit, the DNA template was prepared by digestion of 20 μg pcDNA-Flag-LINC00313 DNA with XhoI at 37°C for 2 h, followed by ethanol precipitation with sodium acetate (pH 5.2). The *in vitro* transcription was performed by incubating 3 μg of linearized DNA template with T7 RNA polymerase at 37°C for 2 h according to the manufacturer’s instructions. The *in vitro*-transcribed RNA was purified using a MEGAclear kit (Ambion; AM1908). TCLs prepared from SLK cells transfected with pcDNA3-Flag or pcDNA3-Flag-Tat for 48 h were incubated with 1 μg of biotin-labeled LINC00313 RNA with rotation at 4°C for 3 h, followed by the addition of streptavidin beads for another 1 h. The RNA-bound protein was detected by immunoblotting using anti-Flag antibody. Three percent of TCLs was used as the input.

### Immunoblotting.

Protein samples in SDS loading buffer were boiled at 95°C for 5 min, loaded, and resolved by SDS-PAGE and transferred onto polyvinylidene difluoride (PVDF) membranes. The membranes were blocked with 5% skimmed milk in 1× TBST (Tris-buffered saline with Tween 20), immunoblotted with anti-Flag–horseradish peroxidase antibody (Sigma; A8592), visualized by using Pierce ECL Western blotting substrate (Thermo Scientific; 34080), and imaged with a luminescence/fluorescence imaging system (Fujifilm; LAS-4000).

## Supplementary Material

Supplemental file 1
