# Supplementary material for: HIV-1 Tat Interacts with a Kaposi’s Sarcoma-Associated Herpesvirus Reactivation-Upregulated Antiangiogenic Long Noncoding RNA, LINC00313, and Antagonizes Its Function
Source: J Virol. 2020 Jan 17;94(3):e01280-19. doi: 10.1128/JVI.01280-19 (PMC7000985; doi:10.1128/JVI.01280-19)
Supplement: Supplemental file 1 [file JVI.01280-19-s0001.pdf]

Figure Legends

Fig S1. Nucleus localization of HIV-Tat in nucleus of SLK cells and KS tissues. (A) Purified Flag-tagged HIV-Tat protein was stained with Coomassie blue. Lane 1, molecular mass marker. Lane 2, beads bound HIV-Tat after elution. Lane 3, eluted HIV-Tat. Lane 4, HIV-Tat after concentration. Flag-Tat is indicated (arrow). (B) Representative images from SLK cells incubated with 0.2, 0.5 and 1 μg/ml of HIV-Tat for 48 hours and then stained for HIV-Tat (green) with an anti-Flag antibody. Nuclei were stained with Hoechst (blue). Scale bar indicate 45 μm. (C) Representative images from SLK cells transiently transfected with pcDNA3-Flag and pcDNA3-Flag-HIV-Tat for 48 hours and stained as described in (B). Scale bar indicate 45 μm. (D) Representative images of IHC-staining for HIV-Tat in high-viral-load HIV-positive KS tissue. HIV-negative KS stained negative for HIV-Tat. Scale bar indicate 20 μm.

Fig S2. The effect of HIV-Tat on cell proliferation and viral production of KSHV lytic reactivated SLK cells. (A) Viability of control and iSLK-BAC16 cells treated with Dox (1 μg/ml) with or without of HIV-Tat (0.2 and 2 μg/ml) for 5 days was assessed by MTT. (B) Supernatants from iSLK-BAC16 cells treated as described in (A) were collected at 96 hrs and the viral titers were determined by analyzing the virion-associated DNA levels using TaqMan qPCR. Data represents mean ± SD.

Fig S3. HIV-Tat dose not regulate the expression of LINC00313 in SLK and iSLK-BAC16 cells. Relative expression level of LINC00313 in control and SLK or iSLK-BAC16 cells treated with HIV-Tat (0.2 μg/ml) for 48 hours was assessed by RT-qPCR. Data represents mean ± SD.

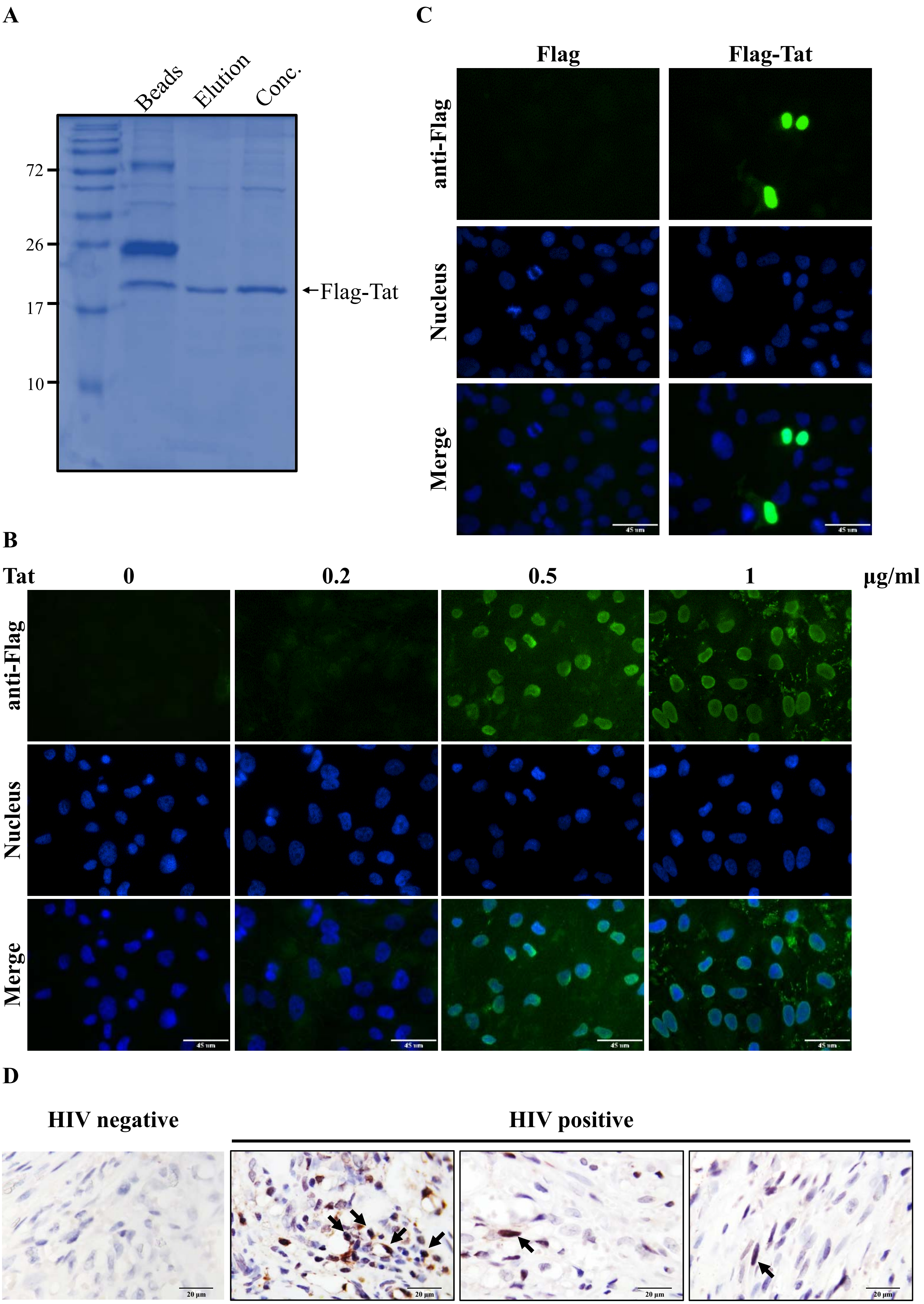
A
Beads
Elution
Conc.
72
26
17
10
Flag-Tat
C
Flag
Flag-Tat
anti-Flag
Nucleus
Merge
B
Tat
0
0.2
0.5
1
µg/ml
anti-Flag
Nucleus
Merge
D
HIV negative
HIV positive
20 µm

A

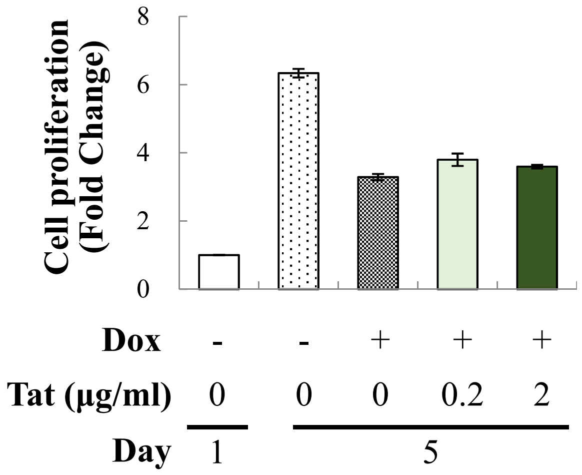
Cell proliferation (Fold Change)
8
6
4
2
0
Dox - - + + +
Tat (µg/ml) 0 0 0 0.2 2
Day 1 5

B

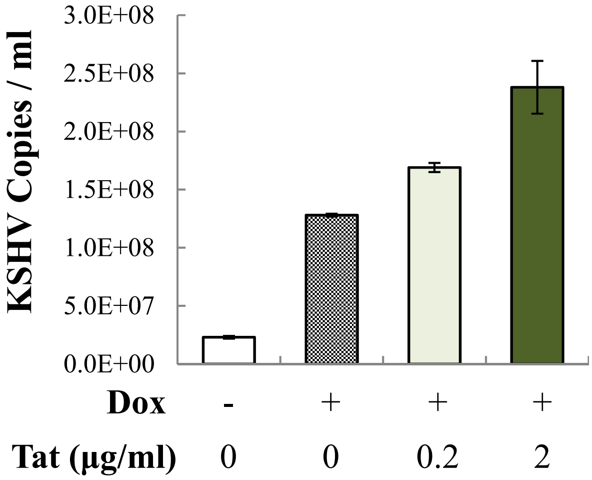
KSHV Copies / ml
3.0E+08
2.5E+08
2.0E+08
1.5E+08
1.0E+08
5.0E+07
0.0E+00
Dox - + + +
Tat (µg/ml) 0 0 0.2 2

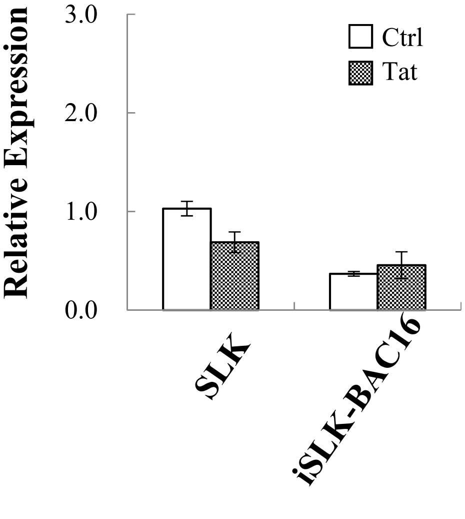
Relative Expression
3.0
2.0
1.0
0.0
Ctrl
Tat
SLK
iSLK-BAC16

TABLE S1 Diseases and functions identified in genes down-regulated by LINC00313 in the absence or presence of HIV-Tat

	**Linc^Mock**	**Tat^Linc**
**Disease and function**	**Z-score**	
Cell movement	-6.38	-
Migration of cells	-5.82	-
Chemotaxis	-4.79	-
Cell movement of blood cells	-4.74	-
Cell movement of leukocytes	-4.47	-
Leukocyte migration	-4.40	-
Mobilization of Ca2+	-4.36	-2.91
Metabolism of reactive oxygen species	-4.13	-2.15
Production of reactive oxygen species	-4.11	-
Homing of blood cells	-4.11	-
Synthesis of reactive oxygen species	-4.03	-
Chemotaxis of leukocytes	-4.00	-
Cell movement of mononuclear leukocytes	-3.98	-
Synthesis of lipid	-3.90	-
Inflammatory response	-3.83	-
Cell movement of myeloid cells	-3.72	-
Chemotaxis of myeloid cells	-3.51	-
Cell movement of granulocytes	-3.49	-
Fatty acid metabolism	-3.40	-
Cell movement of phagocytes	-3.34	-
Interaction of tumor cell lines	-3.34	-
Binding of tumor cell lines	-3.33	-
Chemotaxis of mononuclear leukocytes	-3.29	-
Chemotaxis of granulocytes	-3.23	-
Metabolism of eicosanoid	-2.98	-2.93
Phosphorylation of protein	-2.93	-
Quantity of metal ion	-2.92	-
Cell movement of monocytes	-2.89	-
Differentiation of epithelial cells	-2.80	-
Chemotaxis of phagocytes	-2.80	-
Proliferation of B lymphocytes	-2.76	-
Quantity of Ca2+	-2.76	-
Synthesis of fatty acid	-2.74	-
Differentiation of dermal cells	-2.62	-
Synthesis of eicosanoid	-2.60	-
Non-melanoma solid tumor	-2.58	-
Quantity of cyclic nucleotides	-2.56	-
Cell movement of kidney cell lines	-2.55	-
Cell movement of embryonic cell lines	-2.54	-
Adhesion of epithelial cell lines	-2.50	-
Activation of cells	-2.49	-
Binding of blood cells	-2.43	-
Production of hydrogen peroxide	-2.38	-
Oxidation of hormone	-2.23	-
Ion homeostasis of cells	-2.21	-
Differentiation of epithelial tissue	-2.19	-
Metabolism of retinoid	-2.18	-
Activation of blood cells	-2.10	-
Quantity of blood cells	-2.09	-2.18
Flux of ion	-2.06	-
Synthesis of hormone	-2.04	-
Metabolism of hormone	-2.04	-
Oscillation of Ca2+	-2.00	-
Cell viability of chronic lymphocytic leukemia cells	-2.00	-
